# QuickStats: Age-Adjusted Percentages* of Adults Aged ≥18 Years Who Were Told in the Past 12 Months by a Doctor or Health Professional That They Had Sinusitis,^†^ by Sex, Race, and Hispanic Origin^§^ — National Health Interview Survey, 2017^¶^

**DOI:** 10.15585/mmwr.mm6815a7

**Published:** 2019-04-19

**Figure:**
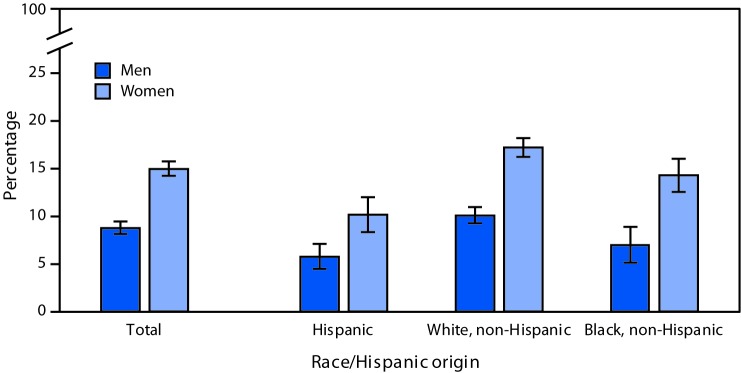
Among adults aged ≥18 years, women (15.0%) were more likely than men (8.8%) to have been told by a doctor or health professional in the past 12 months that they had sinusitis. Among men, non-Hispanic white men (10.1%) were more likely than both non-Hispanic black (7.0%) and Hispanic (5.8%) men to have received a diagnosis of sinusitis. Among women, non-Hispanic white women (17.2%) were most likely to have received a diagnosis of sinusitis, followed by non-Hispanic black (14.3%) and Hispanic (10.2%) women.

**Source:** Tables of Summary Health Statistics, 2017. https://ftp.cdc.gov/pub/Health_Statistics/NCHS/NHIS/SHS/2017_SHS_Table_A-2.pdf.

